# Treatment of stage I-III squamous cell anal cancer: a comparative effectiveness systematic review

**DOI:** 10.1093/jnci/djae195

**Published:** 2024-08-20

**Authors:** Alexander Troester, Romil Parikh, Bronwyn Southwell, Elizabeth Ester, Shahnaz Sultan, Edward Greeno, Elliot Arsoniadis, Timothy R Church, Timothy Wilt, Mary Butler, Paolo Goffredo

**Affiliations:** Department of Surgery, University of Minnesota, Minneapolis, MN, USA; School of Public Health, University of Minnesota, Minneapolis, MN, USA; Department of Anesthesia, University of Minnesota, Minneapolis, MN, USA; Division of Radiation Oncology, Department of Radiology, University of Minnesota, Minneapolis, MN, USA; Division of Gastroenterology, Hepatology, and Nutrition, Department of Medicine, University of Minnesota, Minneapolis, MN, USA; Division of Hematology, Oncology, and Transplantation, Department of Medicine, University of Minnesota, Minneapolis, MN, USA; Division of Colon & Rectal Surgery, Department of Surgery, University of Minnesota, Minneapolis, MN, USA; School of Public Health, University of Minnesota, Minneapolis, MN, USA; Masonic Cancer Center, University of Minnesota, Minneapolis, MN, USA; Minneapolis VA Center for Care Delivery and Outcomes Research and the University of Minnesota Schools of Medicine and Public Health, Minneapolis, MN, USA; School of Public Health, University of Minnesota, Minneapolis, MN, USA; Division of Colon & Rectal Surgery, Department of Surgery, University of Minnesota, Minneapolis, MN, USA

## Abstract

**Background:**

We sought to assess the effectiveness and harms of initial treatment strategies for stage I through III anal squamous cell anal cancer.

**Methods:**

We searched MEDLINE, Embase, and Cochrane Central Register of Controlled Trials between January 1, 2000, and March 2024, for randomized controlled trials and nonrandomized studies of interventions comparing initial treatment strategies. Individual study risk of bias and overall strength of evidence were evaluated for a prespecified outcome list using standardized methods.

**Results:**

We identified 33 eligible studies and extracted data. Six were deemed low to moderate risk of bias. Compared with radiation therapy alone, chemoradiation therapy (CRT) with 5-fluorouracil (5-FU) and mitomycin C probably shows a benefit in locoregional failure, disease-specific survival, and colostomy-free survival (moderate strength of evidence) yet may result in greater overall and acute hematological toxicity, with no difference in late harms (low strength of evidence). CRT with 5-FU plus mitomycin C may show a benefit in locoregional failure, disease-specific survival, and colostomy-free survival rates compared with 5-FU alone (low strength of evidence). CRT with 5-FU plus cisplatin vs 5-FU plus mitomycin C probably results in no differences in several effectiveness outcomes or overall acute or late harms and probably increases hematological toxicity with mitomycin C (moderate strength of evidence). Compared with CRT using capecitabine plus mitomycin C, CRT with capecitabine plus mitomycin C and paclitaxel may improve overall survival, disease-specific survival, and colostomy-free survival yet cause more acute harms (low strength of evidence). Evidence was insufficient for remaining comparisons.

**Conclusions:**

CRT with 5-FU plus mitomycin C or 5-FU plus cisplatin is likely more effective yet incurs greater acute hematological toxicity than radiation therapy alone or single-agent CRT. Adding paclitaxel to capecitabine plus mitomycin C may increase treatment efficacy and toxicity. Evidence is insufficient comparing posttreatment surveillance strategies and patient-reported outcomes, highlighting research opportunities.

Squamous cell carcinoma of the anal canal is an uncommon tumor. Although it represents 1% to 2% of all gastrointestinal malignancies, incidence rates have been rising steadily over the past decade (1,2). Historically managed with abdominoperineal resection and permanent colostomy, in 1974, Nigro and colleagues introduced the “Nigro protocol,” shifting squamous cell anal cancer treatment toward largely medical therapies after demonstrating long-term oncological outcomes similar to abdominoperineal resection without its adverse consequences (3,4). Subsequently, 3 phase III trials established concurrent chemoradiation therapy (CRT) as standard of care (5-7), which consists of 5-fluorouracil (5-FU) and mitomycin C with concomitant radiation between 30 and 45 Gy over 3.5 to 5 weeks, with an additional 5- to 24-Gy boost (8-10). This regimen is highly effective, with a 5-year survival up to 89%, depending on initial staging, and yielding sphincter preservation rates of approximately 80% (2,11).

Despite excellent clinical results with concurrent CRT, these regimens are associated with clinically significant toxicity, with up to 55% of patients requiring treatment breaks. This trend has led to interest in alternative treatments to maintain efficacy while reducing harms and burden (4,11-14). Potentially less toxic protocols under investigation include local excision alone for early-stage disease, administration of capecitabine and/or immunotherapy, use of proton beam radiation therapy (RT), and de-escalation of radiation dosing for appropriate stages (11). Conversely, more advanced tumors are less likely to respond to existing therapeutic approaches; thus, radiation dose escalation remains a topic of investigation for these patients (11). Furthermore, data are scarce regarding the efficacy of different posttreatment surveillance frequencies and modalities because standard of care was established in the unvalidated schedule implemented in the Radiation Therapy Oncology Group (RTOG) 98-11 trial (15,16). Critical appraisal of advances in treatment regimens has been inadequate, with the last randomized controlled trial having been conducted more than a decade ago (12). Thus, recent innovations in the field have largely relied on limited high-quality data to guide clinical decision making.

We conducted a comparative effectiveness evaluation for existing and emerging therapies as well as de-escalation and posttreatment surveillance strategies to identify the optimal management approach that reduces toxicity without compromising oncological outcomes or patient quality of life (QOL). Findings will inform clinical guideline committees as they update recommendations.

## Methods

### Key questions

This systematic review addressed 6 key questions (see Supplementary Methods, available online) that were developed in conjunction with key informants before protocol development, with minor revisions after correspondence with a multidisciplinary technical expert panel (Supplementary Table 1, available online). The key questions were designed to evaluate the effectiveness and harms of different modalities of initial treatment for stage I through III squamous cell anal cancer, including RT delivery (dose, volume, and fractionation schema), chemotherapy and radiation combinations, dose escalation and de-escalation, immunotherapy, and posttreatment surveillance.

### Protocol standards

The methodology outlined in the Agency for Healthcare Research and Quality (AHRQ) Methods Guide for Effectiveness and Comparative Effectiveness Reviews was employed (17). Adherence to reporting standards was ensured by following the Preferred Items for Reporting in Systematic Reviews and Meta-Analyses, A Measurement Tool to Assess Systematic Reviews, and the Patient-Centered Outcomes Research Institute Methodology Standards checklist (18-20). The complete protocol is registered on PROSPERO with the registration No. CRD42023456886 and is publicly available on the Effective Health Care Program website at https://effectivehealthcare.ahrq.gov/products/anal-cancer-treatment/protocol.

### Study selection

A search was conducted using Medline, Embase, and the Cochrane Central Register of Controlled Trials, covering the period from January 1, 2000, to March 2024. The search strategy used controlled vocabulary and natural language terms as well as free-text words. To complement database searches, citation searches were performed on systematic reviews and original research, including all eligible studies irrespective of publication date. The searches were executed by a qualified search specialist and underwent peer review. Inclusion criteria were adults with stage I through III squamous cell anal cancer of either the anal canal or the anal margin that reported oncological, functional, and harms outcomes of interest; a comprehensive list of included studies can be found in Supplementary References (available online). Studies were excluded if they contained mixed populations of individuals with stage I through IV squamous cell anal cancer, where more than 20% of the population was stage IV, patients with lower rectal cancer that spread to the anal canal, or patients with nonsquamous histologies.

The web-based screening tool PICO Portal (www.picoportal.net) was used to upload and screen citations. Duplicate citations were removed using the PICO Portal software. Initially, 2 independent reviewers screened titles and abstracts for relevance to the key questions. Any discrepancies were resolved through group discussions with the review team. Once the machine learning algorithm achieved a predefined 90% recall rate of eligible citations (as outlined in the review protocol), the team transitioned to a single independent reviewer until a 100% recall rate and a zero false-negative rate were attained.

At the full-text level, 2 independent reviewers screened each article using eligibility criteria detailed in Supplementary Table 2 (available online). All available randomized controlled trials for each distinct comparison were included. For other comparisons, we relied on nonrandomized studies of interventions with adequate methodology to evaluate associations. Due to the heterogeneity in harms reporting across studies, definitions within each individual study were used to report harms. In addition, when harms were categorized according to the Common Terminology Criteria for Adverse Events, harm grades 2 through 4 were reported. To assess publication and reporting bias as well as identify ongoing studies, ClinicalTrials.gov was queried for relevant completed studies without published outcomes. Despite issuing a *Federal Register* notice soliciting Supplemental Evidence and Data for Systematic Review, we did not receive any responses.

### Risk-of-bias assessment

Risk of bias was evaluated using the Cochrane Risk of Bias Tool, version 2.0 for randomized controlled trials and the Risk Of Bias In Non-Randomized Studies – of Interventions for nonrandomized studies of interventions (21,22). Randomized controlled trials were categorized as low, moderate, or high risk of bias. Non-randomized studies of interventions were classified as low, moderate, serious, or critical risk of bias based on Risk Of Bias In Non-Randomized Studies – of Interventions criteria. One investigator conducted the initial risk-of-bias assessment, and a second reviewer conducted a subsequent review. Any discrepancies were resolved through team consensus.

### Data extraction and management

Data extraction for all studies, independent of risk-of-bias assessment, was performed using a standardized form in Microsoft Excel. Information extracted included author, year of publication, funding source, study setting, inclusion and exclusion criteria for participants, characteristics of interventions and controls, sample size, follow-up duration, participant baseline demographics, clinical characteristics, outcomes, and adverse effects. One reviewer conducted an initial data extraction, with a second reviewer verifying the accuracy of the extracted data.

### Data synthesis

All included studies were organized based on key question. Within each question, further organization was carried out based on the unique comparison, outcomes analyzed, and its timing. Due to the diversity of interventions across studies and the limited number of studies for each comparison, data pooling was not feasible. Consequently, a qualitative synthesis of the data was performed.

### Evaluation of the strength of evidence

The evaluation of the strength of evidence for outcome-intervention pairs was conducted in accordance with AHRQ methods (23). The overall strength of evidence was assigned a grade of high, moderate, low, or insufficient (Supplementary Table 3, available online). In situations where both randomized controlled trials and post hoc secondary analyses of randomized controlled trials contributed to a particular outcome, primary trials were prioritized over post hoc studies. For bodies of evidence consisting of only a single study, consistency was designated as not applicable. Given the limited amount of high-quality data, especially pertaining to key questions 2 through 6, results from outcome-intervention pairs with insufficient strength of evidence were reported. These results should be interpreted with caution, however, because their inclusion is intended to provide a comprehensive overview for the current state of the literature.

## Results

**Figure 1.**
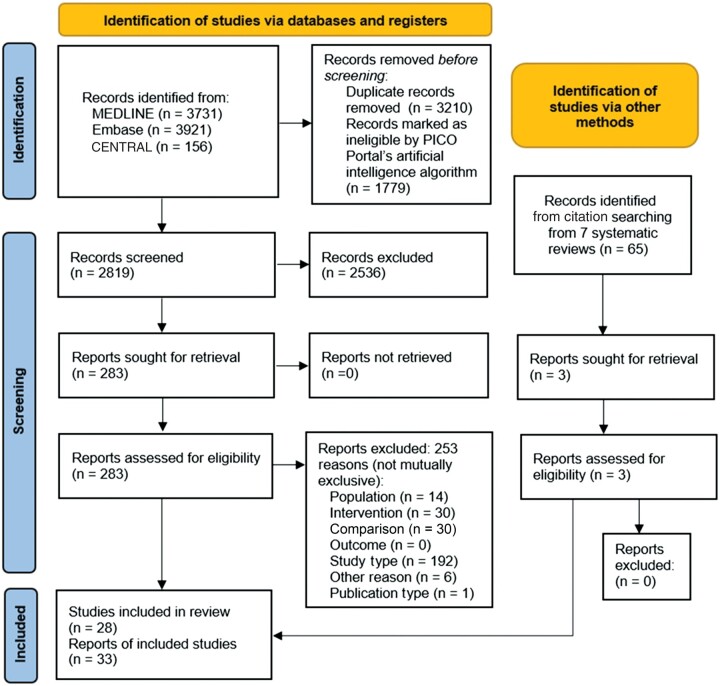
Literature flow diagram. CENTRAL = Cochrane Central Register of Controlled Trials.

The literature search identified 7366 references (Figure 1). Title and abstract screening eliminated 7093 references, leaving 273 references for full-text review. A total of 33 eligible references were identified and their outcomes data extracted. Overall, 8 were unique randomized controlled trials and 20 were nonrandomized studies of interventions. Six randomized controlled trials were deemed low or moderate risk of bias. Supplementary References (available online) contains a list of all eligible publications. Supplementary Table 4 (available online) lists study characteristics. Table 1 summarizes key findings by interventions and outcomes.

**Table 1.** Summary of key findings^a^

Intervention vs comparison	Outcome	No. of studies and design; No. of participants	Findings	Strength of evidence
CRT with 5-FU and mitomycin C vs RT alone (Bartelink, 1997; ACT I, 1996) (5,6)	Overall survival	2 randomized controlled trials; 695	No significant difference 5-y, 58% for CRT vs 54% for RT (*P* = .17, n = 110) 3-y, 65% for CRT vs 58% for RT (relative risk = 0.86, 95% CI = 0.67 to 1.11, *P* = .25, n = 585)	Moderate
	Disease-specific mortality	1 randomized controlled trial; 585	Favors CRT over RT 28% for CRT vs 39% for RT (relative risk = 0.71, 95% CI = 0.53 to 0.95)	Moderate
	Complete response rate (6 wk after treatment)	2 randomized controlled trials; 695	Favors CRT over RT 80% for CRT vs 54% for RT (*P* < .05, n = 110) 39% for CRT vs 30% for RT (*P* < .05, n = 585)	Low
	Locoregional failure rate	2 randomized controlled trials; 695	Favors CRT over RT 5-y, 32% for CRT vs 50% for RT (*P* = .02, n = 110) 3-y, 39% for CRT vs 61% for RT (relative risk = 0.54, 95% CI = 0.42 to 0.69; n = 585)	Moderate
	Colostomy-free survival^b^	1 randomized controlled trial; 110	Favors CRT over RT Improvement of 32% at 5 y (*P* = .002)	Low
	Overall acute harms^c^	1 randomized controlled trial; 585	Significantly greater in CRT Event rate: 47.9% for CRT vs 38.5% for RT	Moderate
	Acute hematological toxicity	2 randomized controlled trials; 695	Significantly greater in CRT Grade 4 events: 1 for CRT vs 0 for RT (n = 110) Overall events: 20 for CRT vs 0 for RT (n = 585)	Low
	Acute dermatological toxicity	2 randomized controlled trials; 695	No significant difference Grade 3+ events: 51.0% for CRT vs 55.8% for RT (*P* > .05, n = 110) Events: 31.8% for CRT vs 26.7% for RT (*P* > .05, n = 585)	Low
	Acute gastrointestinal toxicity	2 randomized controlled trials; 695	No significant difference Grade 3+ events: 19.6% for CRT vs 7.7% for RT (*P* > .05, n = 110) Events: 15.8% for CRT vs 13.7% for RT (*P* > .05, n = 585)	Low
	Acute genitourinary toxicity^c^	1 randomized controlled trial; 585	No significant difference Events: 6.8% for CRT vs 4.6% for RT (*P* > .05)	Low
	Overall late harms^d^	1 randomized controlled trial; 585	No significant difference Event rate: 41.8% for CRT vs 37.9% for RT (*P* = .39)	Low
	Late dermatological toxicity	2 randomized controlled trials; 695	No significant difference Events: 5.9% for CRT vs 3.8% for RT (*P* > .05, n = 110) Events: 20.2% for CRT vs 16.5% for RT (*P* > .05, n = 585)	Low
	Late gastrointestinal toxicity^d^	1 randomized controlled trial; 585	No significant difference Events: 28.8% for CRT vs 27.0% for RT	Low
	Late genitourinary toxicity	1 randomized controlled trial; 585	No significant difference Events: 6.2% for CRT vs 6.7% for RT	Low
CRT with 5-FU and mitomycin C vs 5-FU alone (Flam, 1996) (7)	Overall survival	1 randomized controlled trial; 310	No significant difference 4-y: 78.1% for 5-FU plus mitomycin C vs 71.0% for 5-FU (*P* = .31)	Low
	Complete response rate (4-6 wk after treatment)	1 randomized controlled trial; 310	No significant difference 92.2% for 5-FU plus mitomycin C vs 86.0% for 5-FU (*P* = .14)	Low
	DFS	1 randomized controlled trial; 310	Favors 5-FU plus mitomycin C 4-y: 73% for 5-FU plus mitomycin C vs 51% for 5-FU (*P* < .001)	Low
	Locoregional failure rate	1 randomized controlled trial; 310	Favors 5-FU plus mitomycin C 4-y: 16% for 5-FU plus mitomycin C vs 34% for 5-FU (*P* < .001)	Low
	Colostomy-free survival	1 randomized controlled trial; 310	Favors 5-FU plus mitomycin C 4-y: 71% for 5-FU plus mitomycin C vs 59% for 5-FU (*P* = .014)	Low
CRT with 5-FU and mitomycin C vs 5-FU and cisplatin (James 2013, Ajani 2008) (12,15)	Overall survival	2 randomized controlled trials; 1622	No significant difference HR = 1.28, 95% CI = 0.90 to 1.84, cisplatin (referent), n = 682); median follow-up = 2.5 y HR = 1.05, 95% CI = 0.80 to 1.38; mitomycin C (referent), n = 940; median follow-up = 5.1 y	Moderate
	Distant metastasis	1 randomized controlled trial; 682	No significant difference 15% for mitomycin C vs 19% for cisplatin (*P* = .14); median follow-up = 2.5 y	Moderate
	Locoregional failure	1 randomized controlled trial; 682	No significant difference HR = 1.32, 95% CI = 0.98 to 1.78; cisplatin (referent); median follow-up = 2.5 y	Low
	DFS	1 randomized controlled trial; 682	No significant difference HR = 1.20, 95% CI = 0.93 to 1.55; cisplatin (referent); median follow-up = 2.5 y	Low
	PFS	1 randomized controlled trial; 940	No significant difference HR = 0.95, 95% CI = 0.75 to 1.19; mitomycin C (referent); median follow-up = 5.1 y	Moderate
	Complete response rate (26 wk after treatment)	1 randomized controlled trial; 940	No significant difference 90.5% for mitomycin C vs 89.6% for cisplatin (*P* > .05); median follow-up = 5.1 y	Moderate
	Acute overall harms	2 randomized controlled trials; 1622	No significant difference Grade 3+ events: 87% for mitomycin C vs 83% for cisplatin (*P* = .13, n = 682) Grade 3+ events: 71% for mitomycin C vs 72% for cisplatin (*P* > .05, n = 940)	Moderate
	Acute hematological toxicity	2 randomized controlled trials; 1622	Significantly greater with mitomycin C Grade 3+ events: 61% for mitomycin C vs 42% for cisplatin (*P* < .001, n = 682) Grade 3+ events: 26% for mitomycin C vs 16% for cisplatin (*P* < .001, n = 940)	Moderate
	Acute dermatological toxicity	2 randomized controlled trials; 1622	No significant difference Grade 3+ events: 48% for mitomycin C vs 41% for cisplatin (*P* = .09, n = 682) Grade 3+ events: 48% for mitomycin C vs 47% for cisplatin (*P* > .05, n = 940)	Low
	Acute gastrointestinal toxicity	2 randomized controlled trials; 1622	No significant difference Grade 3+ events: 32% for mitomycin C vs 43% for cisplatin (*P* > .05, n = 682) Grade 3+ events: 16% for mitomycin C vs 18% for cisplatin (*P* > .05, n = 940)	Low
	Acute genitourinary toxicity	2 randomized controlled trials; 1622	No significant difference Grade 3+ events: 3% for mitomycin C vs <1% for cisplatin (*P* > .05, n = 682) Grade 3+ events: 1% for mitomycin C vs 2% for cisplatin (*P* > .05, n = 940)	Low
	Late harms	1 randomized controlled trial; 682	No significant difference, imprecise estimates	Low
CRT with capecitabine plus mitomycin C and paclitaxel vs capecitabine plus mitomycin C (Gordeyev, 2022) (27)	DFS	1 randomized controlled trial; 144	Favors paclitaxel group at 3 y 87.1% for paclitaxel vs 64.4% for control (*P* = .001)	Low
	Overall survival	1 randomized controlled trial; 144	Favors paclitaxel group at 3 y 95.5% for paclitaxel vs 80.0% for control (*P* < .001)	Low
	Colostomy-free survival	1 randomized controlled trial; 144	Favors paclitaxel group at 3 y 83.2% for paclitaxel vs 67.5% for control (*P* = .029)	Low
	Complete response rate (26 wk after treatment)	1 randomized controlled trial; 144	Favors paclitaxel group 88.9% for paclitaxel vs 75.0% for control (*P* = .049)	Low
	Acute overall harms^e^	1 randomized controlled trial; 144	Increased in paclitaxel group 56.9% for paclitaxel vs 26.4% for control (*P* < .001)	Low
	Acute hematological toxicity^e^	1 randomized controlled trial; 144	No significant difference Grade 3+ neutropenia: 16.7% for paclitaxel vs 9.7% for control (*P* = .22)	Low
	Acute gastrointestinal toxicity	1 randomized controlled trial; 144	No significant difference Grade 3+ diarrhea: 13.9% for paclitaxel vs 6.9% for control (*P* = .17) Grade 3+ proctitis: 12.5% for paclitaxel vs 5.6% for control (*P* = .15)	Low

a Remaining comparisons not mentioned in the table received an insufficient grade. 5-FU = 5-fluorouracil; ACT I = Anal Cancer Trial I; CI = confidence interval; CRT = chemoradiation therapy; DFS = disease-free survival; HR = hazard ratio; PFS = progression-free survival; RT = radiation therapy.

b Calculated as a proportion of patients alive at 5 years, with and without a colostomy.

c Harms within 8 weeks after treatment.

d Harms more than 8 weeks after treatment.

e Assessed at 30 days after treatment completion.

### Key question 1: What are the effectiveness and harms of different modalities of initial treatment for stage I through III squamous cell anal cancer?

Two randomized controlled trials (1 low, 1 moderate risk of bias) and 1 nonrandomized studies of intervention (high risk of bias), a post hoc analysis of an included randomized controlled trial, compared concurrent CRT to RT alone (Table 1) (5,6,24). The European Organization for Research and Treatment of Cancer trial (5) and the United Kingdom Co-ordinating Committee on Cancer Research Anal Cancer Trial I (ACT I) (6) delivered RT at 45 Gy, with reassessment at 6 weeks, with a 15-Gy or greater boost for good responders and salvage surgery for poor responders. Although the chemotherapy dosing and schedules of the ACT I trial (5-FU 1000 mg/m^2^ on days 1-4 or 750 mg/m^2^ on days 1-5, and mitomycin C 12 mg/m^2^ on day 1) and European Organization for Research and Treatment of Cancer (5-FU 750 mg/m^2^ on days 1-5 and days 29-33 and mitomycin C 15 mg/m^2^ on day 1) differed slightly, the modalities of delivering RT were similar. Overall, compared with RT alone, concurrent CRT with 5-FU plus mitomycin C improved disease-free survival (DFS), had greater complete response rates, and improved colostomy-free survival up to 5 years (low to moderate strength of evidence), but the effect magnitude varied by study (Table 1). Statistically significant lower 5-year locoregional failure rates were observed in both randomized controlled trials in favor of concurrent CRT, with effects persisting up to 12 years according to the post hoc analysis (hazard ratio [HR] = 0.61, 95% confidence interval [CI] = 0.49 to 0.76) (24). Both randomized controlled trials reported no statistically significant difference in overall survival between CRT and RT alone (moderate strength of evidence). In addition, in the concurrent CRT groups for each randomized controlled trial, there were greater overall acute harms (moderate strength of evidence) but no difference in late harms (low strength of evidence) (Table 1).

Radiation Therapy Oncology Group (RTOG) trial 87-04 (moderate risk of bias) compared CRT with 5-FU alone with a 2-agent regimen of 5-FU plus mitomycin C (7). Compared with 5-FU alone, 5-FU plus mitomycin C showed statistically significant lower locoregional failure rates (16% vs 34%, *P* < .001), improved DFS (73% vs 51%, *P* < .001), and improved colostomy-free survival (71% vs 59%, *P* < .001) (low strength of evidence) (Table 1) (7). There were no statistically significant differences in overall survival (78.1% vs 71%, *P* = .31) or complete response rates (92.2% vs 86%, *P* = .14), with up to 4 years of follow-up (low strength of evidence) (Table 1) (7). Evidence was insufficient to compare harms.

Four reports of 2 randomized controlled trials (low risk of bias for randomized controlled trials, high risk of bias for post hoc analyses) compared CRT with 5-FU plus mitomycin C vs 5-FU plus cisplatin, with important differences in trial design (12,15,25,26). The RTOG 98-11 trial allowed higher doses of radiation as well as mitomycin C and cisplatin than did the ACT II trial (12,15). Moreover, in the RTOG 98-11 trial, only the cisplatin arm had an additional induction chemotherapy phase (15). Noting these variances, there were no statistically significant differences in overall survival between mitomycin C and cisplatin in either randomized controlled trial (12,15) (moderate strength of evidence) and no statistically significant difference in DFS in the RTOG 98-11 trial (low strength of evidence) (Table 1) (15). A post hoc analysis of the RTOG 98-11 trial, however, showed a statistically significant benefit in overall survival (HR = 1.39, 95% CI = 1.05 to 1.78) and DFS (HR = 1.40, 95% CI = 1.11 to 1.78), favoring mitomycin C over cisplatin over a follow-up of 8 years (26). There were no statistically significant differences in locoregional failure (low strength of evidence) or distant metastasis rates (moderate strength of evidence) between mitomycin C and cisplatin (Table 1) (15,26). Colostomy-free survival results were inconsistent, with the ACT II trial and its post hoc analysis reporting no statistically significant differences (12,25), while the RTOG 98-11 trial reported a statistically significant greater colostomy-free survival favoring mitomycin C over cisplatin (15), yet this observed benefit was attenuated and ultimately not statistically significant over a longer follow-up in post hoc analysis (26). A paired regimen with mitomycin C had statistically significant greater severe acute hematological toxicity compared with cisplatin in both randomized controlled trials (moderate strength of evidence) (Table 1).

Finally, 1 recently published phase 3 randomized controlled trial (low risk of bias) examined CRT with capecitabine plus mitomycin C vs capecitabine plus mitomycin C and paclitaxel (27). Study groups were balanced, and the primary endpoint of 3-year DFS saw a statistically significant improvement in the paclitaxel group compared with controls (87.1% vs 64.4%, *P* = .001). Moreover, the paclitaxel group demonstrated better 3-year overall survival (95.5% vs 80%, *P* < .001), 3-year colostomy-free survival (83.2% vs 67.5%, *P* = .03), and clinical complete response at 26 weeks (88.9% vs 75%, *P* = .049). Overall acute harms at 30 days occurred more often in the paclitaxel group (56.9% vs 26.4%, *P* < .001), yet grade 3+ neutropenia (16.7% vs 9.7%, *P* = .22), grade 3+ diarrhea (13.9% vs 6.9%, *P* = .17), and grade 3+ proctitis (12.5% vs 5.6%, *P* = .15) showed no difference (low strength of evidence) (Table 1).

Strength of evidence was insufficient for remaining comparisons under key question 1, including local excision vs CRT for early-stage cancer and capecitabine vs 5-FU. All findings with insufficient strength of evidence are summarized in Supplementary Table 5 (available online).

### Key question 2: What are the effectiveness and harms of different modalities of RT for initial treatment of stage I through III squamous cell anal cancer?

Two nonrandomized studies of interventions compared intensity-modulated RT vs non–intensity-modulated RT, 1 using the Surveillance, Epidemiology, and End Results–Medicare database (28) while the other included individuals from the US Department of Veterans Affairs database (29). In both studies, patients not undergoing intensity-modulated RT could be treated with either 2-dimensional or 3-dimensional conformal RT. Patients receiving intensity-modulated RT were more likely to be treated with mitomycin C than with cisplatin (41% vs 29.7%, *P* = .05) (28) or mitomycin C compared with an alternative chemotherapy regimen (88% vs 72%, *P* < .01) (29). Adjusted analysis at 5 years demonstrated no difference in overall mortality (HR = 0.89, 95% CI = 0.66 to 1.21) or anal cancer mortality (HR = 0.72, 95% CI = 0.45 to 1.17) (30). Serious risk of bias was determined based on missing details for chemotherapy agents used and inadequate adjustments for confounding by different chemotherapy regimens. Thus, strength of evidence was determined to be insufficient, and findings from this comparison are detailed in Supplementary Table 5 (available online).

Intensity-modulated RT was compared with 3-dimensional conformal RT in 2 studies: 1 using the National Cancer Database (30) and 1 a single-center retrospective review (31). The National Cancer Database study had critical risk of bias because of insufficient information about chemotherapy agents and variations in RT protocols, while the single-center review had critical risk of bias because of heterogeneity in CRT protocols, inadequate confounding adjustment, and lack of information about statistical power calculations. Comparisons of overall survival, colostomy-free survival, locoregional recurrence-free survival, and distant metastasis–free survival were determined to have insufficient strength of evidence and are summarized in Supplementary Table 5 (available online).

One multicenter retrospective study examined proton–intensity-modulated RT vs photon–intensity-modulated RT (32). Patients undergoing proton–intensity-modulated RT were more likely to have negative nodal status (proton, 69% vs photon, 43%; *P* = .003). In addition, chemotherapy protocols and RT boost techniques varied, which was not sufficiently addressed by adjustment, leading to a determination of serious risk of bias. Progression-free survival (PFS) was not statistically significant after propensity score weighting (HR = 0.6, 95% CI = 0.4 to 1.1). Additional oncologic and harms outcomes are presented in Supplementary Table 5 (available online).

Finally, 2 publications from the same prospective cohort (33,34) and 1 secondary analysis of the ACT I trial (35) (all 3 with serious risk of bias) compared RT boost with external beam RT vs brachytherapy. The 2 prospective cohort studies had no statistically significant imbalance between the external beam RT and brachytherapy arms, yet they allowed variable chemotherapy protocols and selected some centers where brachytherapy boost was not provided. The subgroup analysis of the ACT I trial lacked information about variations in initial treatment as well as characteristics between the external beam RT and brachytherapy groups. Despite these limitations, at a median follow-up of 13.1 years, overall survival (HR = 1.14, 95% CI = 0.81 to 1.6), disease-specific survival (HR = 1.16, 95% CI = 0.70 to 1.93), and relapse-free survival (HR = 1.26, 95% CI = 0.91 to 1.75) did not differ between external beam RT and brachytherapy boost in the ACT I subgroup analysis. Supplementary Table 5 (available online) documents the remaining outcomes.

### Key question 3: What are the effectiveness and harms of different RT doses, volumes, and fractionation schema for initial treatment of stage I through III squamous cell anal cancer?

Four studies compared different doses of RT (36-39). Two publications from the ACCORD 03 trial analyzed a regimen with a standard-dose boost (45 Gy in 25 fractions over 5 weeks + 15 Gy boost) vs a high-dose boost (45 Gy in 25 fractions over 5 weeks + 20-25 Gy boost) (36,37). One retrospective study compared 45 to 54 Gy against more than 54 Gy using the National Cancer Database (38), while the other nonrandomized studies of interventions was a secondary analysis from the ACT II trial, where the authors compared 6 different combinations of radiation dose and total treatment time (39). The 2 publications from the ACCORD 03 trial had high risk of bias because there was considerable attrition and boost modality varied across arms. The 2 nonrandomized studies of interventions had critical risk of bias for not sufficiently accounting for heterogeneity in CRT protocols. Despite 1 nonrandomized study of interventions showing a statistically significant difference in overall survival (favoring 45-54 Gy over >54 Gy; HR = 1.10, 95% CI = 1.01 to 1.20), the other 3 studies found no statistically significant differences (Supplementary Table 5, available online).

Two nonrandomized studies of interventions evaluated dosimetry-based predictors of acute and late toxicity (Supplementary Table 5, available online) (40,41). Both had critical risk of bias because they had a high potential for selection bias and derived dosimetry-based predictors by using a lowest *P* value approach from the same population in which they further evaluated these selected parameters in multivariable regression models, adjusting for limited confounders, without any external validation.

### Key question 4: What are the effectiveness and harms of different combinations of chemotherapy and RT as well as dose de-escalation or dose escalation for initial treatment of stage I through III squamous cell anal cancer?

Induction chemotherapy with 5-FU plus cisplatin was compared with no induction chemotherapy in 2 publications from the ACCORD 03 trial (36,37). The 2 studies were considered to high risk of bias as a result of considerable attrition leading to underpowered analyses in addition to variable radiation boost modalities (external beam RT or brachytherapy) across arms. Despite these shortcomings, no differences were seen in oncologic outcomes or toxicities, apart from more acute grade 3+ hematological toxicity in the induction chemotherapy arm (insufficient strength of evidence) (Supplementary Table 5, available online).

Similarly, maintenance chemotherapy with 5-FU plus cisplatin was compared with no maintenance chemotherapy in the ACT II trial and was considered to have high risk of bias because of substantial attrition in the maintenance arm alone after randomization and the lack of toxicity data in the no maintenance arm (12). No statistically significant differences were observed in terms of overall survival, PFS, colostomy-free survival, and disease-specific survival between maintenance and no maintenance chemotherapy (insufficient strength of evidence) (Supplementary Table 5, available online).

Receipt of either 1 cycle of mitomycin C or 2 cycles in a concurrent CRT regimen of 5-FU plus mitomycin C was compared in a retrospective study (42). Although CRT protocols were mostly similar, patients receiving 2 cycles of mitomycin C were more likely to receive intensity-modulated RT, as well, and omission of the second cycle of mitomycin C could have been based on the patient’s health status or physician’s discretion because it was not specified. Although authors reported no statistically significant differences in overall survival, PFS, colostomy-free survival, disease-specific survival, or acute and late toxicities, we determined that there was insufficient strength of evidence for each of these outcomes (Supplementary Table 5, available online).

Finally, RT boost vs no boost was compared in a secondary analysis of the ACT I trial within a subgroup of patients who responded to initial treatment (CRT or RT alone) (35). Boost was either external bean RT (15 Gy), given to 59% of patients, or iridium-192 implant (25 Gy) given to 28% of patients, with 11% of good responders not receiving a boost. When comparing RT boost with no boost, there was no difference in overall survival (HR = 0.74, 95% CI = 0.48 to 1.15), recurrence-free survival (HR = 0.80, 95% CI = 0.52 to 1.22) or locoregional relapse (HR = 0.90, 95% CI = 0.48 to 1.68) (insufficient strength of evidence) (Supplementary Table 5, available online). Importantly, the study did not report characteristics for its included patient sample and thus was deemed to be at serious risk of bias.

### Key question 5: What are the effectiveness and harms of immunotherapy for initial treatment of stage I through III squamous cell anal cancer?

No studies evaluated immunotherapy for initial treatment of squamous cell anal cancer.

### Key question 6: What are the effectiveness and harms of different frequencies and modalities for posttreatment surveillance strategies after initial treatment of stage I through III squamous cell anal cancer?

Posttreatment surveillance event frequencies were compared in 1 single-institutional study (critical risk of bias) of patients with biopsy-proven, nonmetastatic squamous cell anal cancer who underwent CRT using intensity-modulated RT (16). Patients were categorized as low risk (T1-3N0 disease) or high risk (T4N0 or T1-4N+ disease) and followed up every 3 months for 2 years, every 6 months in years 3 through 5, then yearly thereafter. Digital rectal examination; inguinal node palpation; anoscopy; and annual chest, abdominal, and pelvic computed tomographs were conducted per National Comprehensive Cancer Network recommendations (10). Oncological outcomes and toxicities were recorded at each time interval and compared between groups (insufficient strength of evidence) (Supplementary Table 5, available online). This study had variable follow-up time, lacked adjustment for confounding, and had no formal statistical hypothesis testing.

## Discussion

This systematic review assessed the evidence for initial treatment regimens of nonmetastatic (stage I-III) squamous cell anal cancer.

### Chemotherapy regimens

CRT is the primary treatment for patients with stage I through III squamous cell anal cancer, largely established based on findings from the European Organization for Research and Treatment of Cancer, ACT I, and RTOG 87-04 trials (5-7). Although treatment approaches vary widely and the optimal regimen has yet to be determined, current recommendations support the use of combined-modality therapy with fluoropyrimidine (either infused 5-FU or its oral prodrug, capecitabine) along with mitomycin C or cisplatin and radiation (9,10,43). In addition, treatment-related morbidity is considerable yet poorly captured, with inconsistent definitions delineating acute vs late toxicities. Although we identified no high-strength evidence to support an optimal treatment regimen, our review supported that 2-agent chemotherapy with either 5-FU plus mitomycin C or 5-FU plus cisplatin may have greater DFS and colostomy-free survival along with lower locoregional failure rates than single-agent chemotherapy but little to no difference in overall survival or complete response rates. Mitomycin C, when compared with cisplatin in a 2-agent chemotherapy regimen, had greater acute hematological toxicity but similar overall acute harms in both the RTOG 98-11 and ACT II trials. Despite this finding, mitomycin C has historically been favored over cisplatin, based mainly on long-term results of the RTOG 98-11 trial, a preference that merits a more nuanced discussion. The long-term follow-up of RTOG 98-11 was a retrospective post hoc analysis not prespecified in the trial protocol, description of long-term outcome ascertainment was inadequate, and it lacked a thorough competing risks analysis and adjustment for multiple comparisons testing. In addition, the parent study and long-term follow-up showed conflicting results, with colostomy-free survival improving significantly with mitomycin C in the parent trial but not in the long-term follow-up study and vice versa for overall survival and DFS (15,26). Moreover, in RTOG 98-11, only the cisplatin arm had an additional induction phase delivered before concurrent CRT, which could have played a dominant role on the outcomes in this study and limits the ability to draw inferences for an exclusively concurrent CRT regimen. The subsequent ACT II trial was deemed null because it failed to demonstrate an oncological benefit for cisplatin over mitomycin C, with similar toxicity profiles (12). Based on the results of these 2 trials, the main difference between mitomycin C and cisplatin appears to be driven by mitomycin C’s worse hematological toxicity profile. In addition, the ACT II trial was the only study that compared maintenance chemotherapy with no maintenance chemotherapy. In this study, not only was there 20% attrition in the maintenance chemotherapy arm before starting the maintenance regimen but only 44% of the individuals randomly assigned to the maintenance chemotherapy arm completed the regimen without any significant improvement in outcomes. The investigators attributed this high attrition rate to toxicity events or patient preferences without reporting detailed statistics. Thus, the differential attrition poses a risk of bias for estimating the effect of the intervention on the prespecified trial endpoints (overall survival, DSS, PFS, and colostomy-free survival), and our group concluded that the evidence was insufficient.

Another comparison of interest is capecitabine (an orally administered prodrug of 5-FU) vs 5-FU, which is administered as a continuous intravenous infusion for 4 days in weeks 1 and 5 (44). Capecitabine might be a more convenient, pragmatic, and cheaper alternative to infused 5-FU. Our review did not find sufficient direct evidence comparing the effectiveness and harms of these 2 agents, but published literature in advanced gastric and colorectal cancers suggests that capecitabine is noninferior to infused 5-FU (45,46), and some clinical experts recommend the use of either capecitabine or 5-FU in the treatment of nonmetastatic squamous cell anal cancer (10). Finally, although paclitaxel has been incorporated into initial treatment strategies for other squamous cell malignances—namely, locally advanced head and neck cancer (47,48) as well as esophageal cancer (49)—its efficacy has not been adequately evaluated for nonmetastatic squamous cell anal cancer. The International Multicentre Study in Advanced Anal Cancer Comparing Cisplatin Plus 5 FU vs Carboplatin Plus Weekly Paclitaxel (InterAAct; ClinicalTrials.gov identifier NCT02051868) trial included 10% of its population with nonmetastatic squamous cell anal cancer; in addition, a case series of 8 patients from Wisconsin with localized squamous cell anal cancer who were ineligible for 5-FU plus mitomycin C received a carboplatin plus paclitaxel CRT regimen, resulting in a 100% complete clinical response (45,50). Although initial results are promising, further trials are warranted before including paclitaxel in guideline recommendations (27).

Therefore, despite the vast amount of low-quality data available, no chemotherapy regimen has clearly emerged as superior in terms of maximizing oncological outcomes while minimizing treatment-related toxicities. As clinicians counsel patients on their individualized treatment approach, consideration must be given to co-morbid conditions, compliance, cost, drug availability, and implications for QOL.

For all other interventions and outcomes, we found the evidence to be insufficient.

### Radiation therapy

A pivotal principle of RT for squamous cell anal cancer lies in the delivery of a high total fractionated dose without prolongation of overall treatment time because extended treatment time has a detrimental impact on local control (51). Attempts to refine targeted delivery to diseased tissue while simultaneously limiting morbidity to nearby organs at risk have manifested in a shift toward increased use of intensity-modulated RT after the phase II, single-arm RTOG 0529 landmark study was among the first to report clinically acceptable effectiveness and toxicity outcomes in support of intensity-modulated RT (52). Designed to assess grade 2+ combined acute gastrointestinal and genitourinary adverse events using intensity-modulated RT with 5-FU plus mitomycin C compared with the 3-dimensional conformal RT with 5-FU plus mitomycin C arm of RTOG 98-11, RTOG 0529 demonstrated similar rates of such adverse events to RTOG 98-11 (77% in both studies). When compared with RTOG 98-11, however, RTOG 0529 reported a statistically significant reduction in acute grade 2+ hematological (73% vs 85%, *P* = .03), grade 3+ gastrointestinal (21% vs 36% *P* = .01), and grade 3+ dermatological (23% vs 49%, *P* < .01) adverse events. Despite these assumed benefits, our review found no evidence of a difference between intensity-modulated RT and non–intensity-modulated RT techniques in terms of overall survival, disease-specific survival, distant metastasis–free survival, or acute toxicity. Notably, because many of these studies were conducted before the widespread implementation of intensity-modulated RT, the findings in this report may not be fully applicable, especially related to harms, to current practice.

Further efforts to reduce radiation doses to nearby organ systems led to the comparison of intensity-modulated proton therapy and traditional intensity-modulated RT (using photons) after a feasibility study showed similar toxicity rates of intensity-modulated proton therapy to historical controls in the RTOG 0529 trial (53). In line with the prior feasibility study, the 1 nonrandomized study of interventions in this review comparing intensity-modulated proton therapy with intensity-modulated RT found no differences in acute or late toxicity outcomes (32).

Other modifications to RT delivery have been tested. Previously, conventional practices for radiation delivery included split-course therapy, consisting of an initial total dose of 45 Gy to the pelvis, followed by a boost dose of 15 to 20 Gy to the anal canal (after a 6-week to 8-week gap) delivered by either external beam RT or brachytherapy (43). The post hoc analysis of the ACT I trial demonstrated that locoregional control (HR = 0.91, 99% CI = 0.50 to 1.65) and overall survival (HR = 0.74, 99% CI = 0.48 to 1.13) did not differ between patients who received a boost vs patients who did not, although a trend toward improved overall survival was noted in boosted patients (*P* = .06) (35). The same study found no survival differences between external beam RT and brachytherapy, although brachytherapy had a higher incidence of late ulcers and radionecrosis (35). Finally, in the ACCORD 03 trial, no differences were observed in survival outcomes or QOL when comparing standard-dose boost (15 Gy) with high-dose boost (20-25 Gy), although there was a trend toward improved colostomy-free survival in high-dose boost patients (*P* = .067) (36,37). Despite a trend toward improved overall survival, these results call into question the utility of boost therapy, balancing oncological benefit with QOL outcomes that are poorly captured or absent. Further challenging the discussion regarding boost therapy efficacy, contemporary practices favor a course of continuous treatment without a midtreatment break because extended treatment time has a detrimental impact on local control. Therefore, assessing the impact of boost therapy within a split-course regimen represents a limitation of prior work, such that boost therapy effectiveness remains an unanswered question. In addition to boost therapy, varying dose fractionation schemes were assessed regarding whether de-escalation was appropriate in achieving similar survival rates. Our review demonstrated that total radiation doses under 48.6 Gy, 50.4 Gy delivered over more than 42 days, and 4.72 or fewer fractions per week were associated with lower overall survival independent of disease stage (54).

We found that current evidence is insufficient for assessing the optimal method of radiation delivery, presence or absence of a boost, and fractionation scheme. Qualitative outcomes such as poor QOL and worsening sexual function are potential adverse effects of CRT (55,56). The ACCORD 03 trial evaluated QOL, but responses to the QOL survey tools were missing in about two-thirds of the patients, leading to a high risk of bias and insufficient evidence (38). None of the included studies evaluated sexual function.

Noting these significant limitations, our review supports the use of established fractionation schema delivering at least 50.4 Gy over 38 to 42 days and calls into question the use of boost therapy, whether delivered by external beam RT or brachytherapy.

### Posttreatment surveillance

Alongside improving chemotherapeutic development and radiation delivery, optimizing a posttreatment surveillance schedule bridges the gap between therapeutic innovation and health-care implementation. Timely detection of local recurrence, distant metastases, and toxicities allows for appropriate refinement of the treatment regimen. Risk-stratifying tumor characteristics and determining posttreatment relapse patterns could further improve already favorable long-term outcomes. One study included in this review found that 89% of local recurrences occurred by year 2, with the majority being found because of symptoms of anal pain, bleeding, and persistent ulceration, while the remaining were found by a 3-month posttreatment positron emission tomography scan (16). Although the data on surveillance are limited, this finding suggests that adherence to surveillance regimens in the first 2 years is paramount, with the potential for de-escalation.

### Future research

Seven potentially applicable trials identified from the ClinicalTrials.gov registry and 2 trials identified by hand search are expected to provide relevant information based on the key questions and outcomes of interest detailed in this report. Regarding radiation delivery techniques and fractionation schema, 1 trial is comparing photon-based RT (intensity-modulated RT, volumetric modulated arc therapy and helical tomotherapy) with intensity-modulated proton therapy, and 3 trials are assessing different radiation fractionation schema under the heading Personalising Anal Cancer Radiotherapy Dose Protocol as well as Lower-Dose Chemoradiation in Treating Patients With Early-Stage Anal Cancer, the DECREASE Study (ClinicalTrials.gov identifier NCT04166318). Together, these 3 trials will evaluate RT dose escalation and de-escalation strategies in specific clinical scenarios. Three studies will evaluate the role of immunotherapy (nivolumab, sintilimab, and durvalumab) plus CRT vs CRT alone. Finally, 1 trial will examine the role of circulating tumor DNA in follow-up.

Suggested areas for future research include studies on the long-term effectiveness of mitomycin C vs cisplatin; validation of paclitaxel inclusion in initial treatment regimens; local excision vs CRT or de-escalation with RT alone for stage I squamous cell anal cancer; and well-designed nonrandomized studies of interventions to evaluate long-term functional outcomes, QOL, harms, and biomarkers such as circulating tumor DNA or radiologic predictors of response to therapy such that treatment regimens are further tailored to each individual. Focus should be placed on enhancing the quality of large database collections, with attention to individual chemotherapy regimen, surveillance strategies to enhance recurrence detection, and toxicity data to allow for more robust and methodologically sound comparisons. Finally, emphasis should be placed on evaluating the impact of squamous cell anal cancer on underrepresented populations (racial and ethnic minority groups, immunocompromised status) because only scarce, low-quality data were available.

### Limitations

The main limitations in our findings are the quantity and quality of much of the research. We found no more than 3 studies per unique intervention-outcome comparison, and most of the studies had a high risk of bias. We required studies to include an active comparator and more than 15 participants per study arm to better ascertain direct effects of the described interventions. Although we included evidence from studies with higher risk of bias in our review because of the paucity of quality evidence in the current body of literature, caution must be used in applying these conclusions to clinical practice.

Specifically, the evidence regarding interventions for nonmetastatic squamous cell anal cancer is limited in the following ways: 1) Studies comparing chemotherapy regimens used diverse agents and incorporated important treatment heterogeneity within each study; 2) many studies of CRT effectiveness did not include an active comparator arm and so were not included; and 3) nonrandomized trials, particularly database studies, had varying outcome definitions and ascertainment methods as well as suboptimal control of confounders known to affect treatment selection and outcomes. Patients with immunocompromised status, patients ≥65 years of age, and patients from racial or ethnic minority groups were underrepresented in available research, limiting the generalizability of findings.

The lack of moderate-strength to high-strength evidence makes widespread dissemination of several interventions analyzed in this review challenging and leaves patients, their families, and practitioners without definitive answers. Patients with immunocompromised status, older patients, and possibly patients from racial or ethnic minority groups were underrepresented; thus, assessment of outcomes in these populations relied on evidence derived from other groups of individuals. When deciding on treatment approaches to squamous cell anal cancer, institutional protocols and policymakers as well as clinicians will continue to depend on limited amounts of low-strength and moderate-strength evidence, along with subjective observations. For patients diagnosed with nonmetastatic squamous cell anal cancer, interventions should continue to be implemented according to established standards of care while taking individual patient characteristics into consideration.

Although the quantity and methodological quality of evidence are limited, concurrent CRT with 5-FU plus mitomycin C may be more effective but cause greater acute harms than RT alone or CRT plus 5-FU with or without cisplatin for the initial treatment of stage I through III squamous cell anal cancer. Few high-quality data exist comparing radiation delivery techniques, posttreatment surveillance, and the applicability of current interventions across subpopulations, highlighting future research needs. The current evidence serves as a foundation for proposing specific components to be included in future interventions and a defined set of outcome measures to evaluate effectiveness and harms.

## Supplementary Material

djae195_Supplementary_Data

## Data Availability

All data used to inform this manuscript are publicly available.
