# Supplementary material for: Treatment of stage I-III squamous cell anal cancer: a comparative effectiveness systematic review
Source: J Natl Cancer Inst. 2024 Aug 20;117(2):240–52. doi: 10.1093/jnci/djae195 (PMC11807441; doi:10.1093/jnci/djae195)
Supplement: djae195_Supplementary_Data [file djae195_supplementary_data.pdf]

Supplementary Materials

Supplementary Methods. Key Questions

1. What are the effectiveness and harms of different modalities of initial treatment for stages I-III squamous cell anal cancer?
2. What are the effectiveness and harms of different modalities of radiation therapy for initial treatment of stages I-III squamous cell anal cancer?
3. What are the effectiveness and harms of different radiation therapy doses, volumes, and fractionation schema for initial treatment of stages I-III squamous cell anal cancer?
4. What are the effectiveness and harms of different combinations of chemotherapy and radiation therapy, and dose de-escalation or dose escalation for initial treatment of stages I-III squamous cell anal cancer?
5. What are the effectiveness and harms of immunotherapy for initial treatment of stages I-III squamous cell anal cancer?
6. What are the effectiveness and harms of different frequencies and modalities for post-treatment surveillance strategies after initial treatment of stages I-III squamous cell anal cancer?

Supplementary References. Eligible Studies

1. Chai CY, Tran Cao HS, Awad S, Massarweh NN. Management of Stage I Squamous Cell Carcinoma of the Anal Canal. *JAMA surgery.* 2018;153(3):209-215.
2. Deshmukh AA, Zhao H, Das P, et al. Clinical and Economic Evaluation of Treatment Strategies for T1N0 Anal Canal Cancer. *American Journal of Clinical Oncology: Cancer Clinical Trials.* 2018;41(7):626-631.
3. Gao X, Goffredo P, Kahl AR, Charlton ME, Weigel RJ, Hassan I. Chemoradiation versus local excision in treatment of stage I anal squamous cell carcinoma: A population-based analysis. *European journal of surgical oncology : the journal of the European Society of Surgical Oncology and the British Association of Surgical Oncology.* 2020;46(9):1663-1667.
4. Bartelink H, Roelofsen F, Eschwege F, et al. Concomitant radiotherapy and chemotherapy is superior to radiotherapy alone in the treatment of locally advanced anal cancer: results of a phase III randomized trial of the European Organization for Research and Treatment of Cancer Radiotherapy and Gastrointestinal Cooperative Groups. *J Clin Oncol.* 1997;15(5):2040-2049.
5. Epidermoid anal cancer: results from the UKCCCR randomised trial of radiotherapy alone versus radiotherapy, 5-fluorouracil, and mitomycin. UKCCCR Anal Cancer Trial Working Party. UK Co-ordinating Committee on Cancer Research. *Lancet.* 1996;348(9034):1049-1054.
6. Northover J, Glynne-Jones R, Sebag-Montefiore D, et al. Chemoradiation for the treatment of epidermoid anal cancer: 13-year follow-up of the first randomised UKCCCR Anal Cancer Trial (ACT I). *Br J Cancer.* 2010;102(7):1123-1128.

7. Flam M, John M, Pajak TF, et al. Role of mitomycin in combination with fluorouracil and radiotherapy, and of salvage chemoradiation in the definitive nonsurgical treatment of epidermoid carcinoma of the anal canal: results of a phase III randomized intergroup study. *J Clin Oncol.* 1996;14(9):2527-2539.
8. Goodman KA, Julie D, Cercek A, et al. Capecitabine With Mitomycin Reduces Acute Hematologic Toxicity and Treatment Delays in Patients Undergoing Definitive Chemoradiation Using Intensity Modulated Radiation Therapy for Anal Cancer. *Int J Radiat Oncol Biol Phys.* 2017;98(5):1087-1095.
9. Jones CM, Adams R, Downing A, et al. Toxicity, Tolerability, and Compliance of Concurrent Capecitabine or 5-Fluorouracil in Radical Management of Anal Cancer With Single-dose Mitomycin-C and Intensity Modulated Radiation Therapy: Evaluation of a National Cohort. *Int J Radiat Oncol Biol Phys.* 2018;101(5):1202-1211.
10. Peixoto RDA, Wan DD, Schellenberg D, Lim HJ. A comparison between 5-fluorouracil/mitomycin and capecitabine/mitomycin in combination with radiation for anal cancer. *J Gastrointest Oncol.* 2016;7(4):665-672.
11. Matzinger O, Roelofsen F, Mineur L, et al. Mitomycin C with continuous fluorouracil or with cisplatin in combination with radiotherapy for locally advanced anal cancer (European Organisation for Research and Treatment of Cancer phase II study 22011-40014). *Eur J Cancer.* 2009;45(1):2782-2791.
12. Ajani JA, Winter KA, Gunderson LL, et al. Fluorouracil, mitomycin, and radiotherapy vs fluorouracil, cisplatin, and radiotherapy for carcinoma of the anal canal: A randomized controlled trial. *JAMA.* 2008;299(1):1914-1921.

13. Gunderson LL, Winter KA, Ajani JA, et al. Long-term update of US GI intergroup RTOG 98-11 Phase III trial for anal carcinoma: Survival, relapse, and colostomy failure with concurrent chemoradiation involving fluorouracil/mitomycin versus fluorouracil/cisplatin. *J Clin Oncol.* 2012;30(3):4344-4351.

14. James RD, Glynne-Jones R, Meadows HM, et al. Mitomycin or cisplatin chemoradiation with or without maintenance chemotherapy for treatment of squamous-cell carcinoma of the anus (ACT II): a randomised, phase 3, open-label, 2× 2 factorial trial. *The lancet oncology.* 2013;14(6):516-524.

15. Glynne-Jones R, Kadalayil L, Meadows HM, et al. Tumour- and treatment-related colostomy rates following mitomycin C or cisplatin chemoradiation with or without maintenance chemotherapy in squamous cell carcinoma of the anus in the ACT II trial. *Annals of oncology : official journal of the European Society for Medical Oncology / ESMO.* 2014;25(8):1616-1622.

16. Peiffert D, Tournier-Rangeard L, Gerard J-P, et al. Induction chemotherapy and dose intensification of the radiation boost in locally advanced anal canal carcinoma: Final analysis of the randomized UNICANCER ACCORD 03 trial. *J Clin Oncol.* 2012;30(1):1941-1948.

17. Tournier-Rangeard L, Mercier M, Peiffert D, et al. Radiochemotherapy of locally advanced anal canal carcinoma: Prospective assessment of early impact on the quality of life (randomized trial ACCORD 03). *Radiother Oncol.* 2008;87(3):391-397.

18. Wegner RE, Abel S, Hasan S, et al. Trends in radiation dose and technique for anal canal squamous cell carcinoma. *American Journal of Clinical Oncology: Cancer Clinical Trials.* 2019;42(6):519-526.

19. Lukovic J, Hosni A, Liu A, et al. Evaluation of dosimetric predictors of toxicity after IMRT with concurrent chemotherapy for anal cancer. *Radiotherapy and oncology : journal of the European Society for Therapeutic Radiology and Oncology.* 2023;178:109429.
20. Nilsson MP, Gunnlaugsson A, Johnsson A, Scherman J. Dosimetric and Clinical Predictors for Acute and Late Gastrointestinal Toxicity Following Chemoradiotherapy of Locally Advanced Anal Cancer. *Clinical oncology (Royal College of Radiologists (Great Britain)).* 2022;34(1):e35-e44.
21. Mehta S, Ramey SJ, Kwon D, et al. Impact of radiotherapy duration on overall survival in squamous cell carcinoma of the anus. *J Gastrointest Oncol.* 2020;11(2):277-290.
22. Dasgupta T, Rothenstein D, Chou JF, et al. Intensity-modulated radiotherapy vs. conventional radiotherapy in the treatment of anal squamous cell carcinoma: a propensity score analysis. *Radiotherapy and oncology : journal of the European Society for Therapeutic Radiology and Oncology.* 2013;107(2):189-194.
23. Elson JK, Kachnic LA, Kharofa JR. Intensity-modulated radiotherapy improves survival and reduces treatment time in squamous cell carcinoma of the anus: A National Cancer Data Base study. *Cancer.* 2018;124(2):4383-4392.
24. Pollom EL, Wang G, Harris JP, et al. The Impact of Intensity Modulated Radiation Therapy on Hospitalization Outcomes in the SEER-Medicare Population With Anal Squamous Cell Carcinoma. *Int J Radiat Oncol Biol Phys.* 2017;98(1):177-185.
25. Bryant AK, Huynh-Le M-P, Simpson DR, Mell LK, Gupta S, Murphy JD. Intensity Modulated Radiation Therapy Versus Conventional Radiation for Anal Cancer in the Veterans Affairs System. *Int J Radiat Oncol Biol Phys.* 2018;102(1):109-115.

26. Mohiuddin JJ, Jethwa KR, Grandhi N, et al. Multi-institutional Comparison of Intensity Modulated Photon Versus Proton Radiation Therapy in the Management of Squamous Cell Carcinoma of the Anus. *Adv Radiat Oncol.* 2021;6(5):100744.
27. Glynne-Jones R, Sebag-Montefiore D, Adams R, et al. "Mind the gap"--the impact of variations in the duration of the treatment gap and overall treatment time in the first UK Anal Cancer Trial (ACT I). *Int J Radiat Oncol Biol Phys.* 2011;81(5):1488-1494.
28. Hannoun-Levi J-M, Ortholan C, Resbeut M, et al. High-dose split-course radiation therapy for anal cancer: outcome analysis regarding the boost strategy (CORS-03 study). *Int J Radiat Oncol Biol Phys.* 2011;80(3):712-720.
29. Moureau-Zabotto L, Ortholan C, Hannoun-Levi J-M, et al. Role of brachytherapy in the boost management of anal carcinoma with node involvement (CORS-03 study). *Int J Radiat Oncol Biol Phys.* 2013;85(3):e135-142.
30. White EC, Goldman K, Aleshin A, Lien WW, Rao AR. Chemoradiotherapy for squamous cell carcinoma of the anal canal: Comparison of one versus two cycles mitomycin-C. *Radiotherapy and oncology : journal of the European Society for Therapeutic Radiology and Oncology.* 2015;117(2):240-245.
31. Glynne-Jones R, Meadows HM, Lopes A, Muirhead R, Sebag-Montefiore D, Adams R. Impact of compliance to chemoradiation on long-term outcomes in squamous cell carcinoma of the anus: results of a post hoc analysis from the randomised phase III ACT II trial. *Annals of oncology : official journal of the European Society for Medical Oncology.* 2020;31(1):1376-1385.

32. Frazer ML, Yang G, Felder S, et al. Determining Optimal Follow-up for Patients with Anal Cancer following Chemoradiation. *American Journal of Clinical Oncology: Cancer Clinical Trials*. 2020;43(5):319-324.

33. Gordeev SS, Naguslaeva AA, Chernykh MV, et al. The addition of paclitaxel in chemoradiotherapy of anal squamous cell carcinoma: a prospective randomized phase 3 trial. Koloproktologiâ. 2022;21(4):30-38. doi:https://doi.org/10.33878/2073-7556-2022-21-4-30-38.

Supplementary Table 1. Key Informant and Technical Expert Panel Members

Name/Credentials	Affiliation/Institution	Role/Area of Expertise
Paul Romesser, MD	Memorial Sloan Kettering Cancer Center, New York, NY	Radiation Oncology
Scott Steele, MD, MBA	Cleveland Clinic, Cleveland, OH	Colon & Rectal Surgery
Van K. Morris, MD	M.D. Anderson Cancer Center, University of Texas, Houston, TX	Gastrointestinal Medical Oncology
Cathy Eng, MD	Ingram Cancer Center, Vanderbilt University, Nashville, TN	Hematology and Medical Oncology
Brian Czito, MD	Duke Cancer Center, Durham, NC	Radiation Oncology
Mary Feng, MD	UCSF Cancer Center, San Fransisco, CA	Radiation Oncology
Justine Almada, BS*	Co-founder of The Anal Cancer Foundation	Patient advocate and public speaker

*Part of the Key Informant correspondence only. The remaining individuals were members of both the Key Informants and Technical Expert Panel.

Supplementary Table 2. Study eligibility criteria.

PICOTS; KQ	Inclusion Criteria	Exclusion Criteria
**Population; All KQs**	Adults with stages I-III squamous cell anal cancer (anal canal and anal margin)<br>Inclusive of race/ethnicity, sex, immunocompromised status, or other characteristics associated with health inequities	Adults with stage IV anal cancer, lower rectal cancer that has spread to the anal canal, non-squamous histologies (e.g., adenocarcinoma)<br>Studies including mixed populations with Stages I-IV squamous cell anal cancer which contain 20% or greater proportion of stage IV squamous cell anal cancer
**Interventions; KQ 1**	Surgery, radiation therapy, or chemotherapy, alone or in combination as neoadjuvant/ adjuvant or as induction/ maintenance	Reconstructive surgery, palliative therapy (includes chemotherapy with palliative intent), or treatment for premalignant lesions
**Comparison; KQ 1**	Surgery, radiation therapy, or chemotherapy, alone or in combination as neoadjuvant/ adjuvant or as induction/ maintenance	Reconstructive surgery, palliative therapy (includes chemotherapy with palliative intent), or treatment for premalignant lesions
**Interventions; KQ 2**	Different modalities of radiation therapy such as, but not limited to, IMRT, proton radiation therapy, and brachytherapy boost.	Palliative therapy
**Comparison; KQ 2**	Comparators for different modalities of radiation therapy such as, but not limited to, 3DCRT, photon or electron radiation therapy, and external beam radiation therapy boost.	Palliative therapy
**Interventions; KQ 3**	Radiation therapy**:** varying doses, target (primary and nodal) volumes, and fractionation schema	Palliative therapy
**Comparison; KQ 3**	Radiation therapy**:** varying doses, target (primary and nodal) volumes, and fractionation schema	Palliative therapy
**Interventions; KQ 4**	Chemotherapy and radiation therapy combinations (e.g., 5-Fluorouracil, Mitomycin-C, Cisplatin)**:** variations in dose of chemotherapy or radiation therapy	Palliative therapy
**Comparison; KQ 4**	Chemotherapy and radiation therapy combinations (e.g., 5-Fluorouracil, Mitomycin-C, Cisplatin): variations in dose of chemotherapy or radiation therapy	Palliative therapy
**Interventions; KQ 5**	Immunotherapy (e.g., pembrolizumab, nivolumab)	
**Comparison; KQ 5**	Other treatment (e.g., chemotherapy, radiation therapy, chemotherapy + radiation therapy)	

PICOTS; KQ	Inclusion Criteria	Exclusion Criteria
**Interventions; KQ 6**	Post-treatment surveillance strategies**:** variations in frequency and in modalities (e.g., CT, MRI, PET scans, biopsy, DRE, anoscopy, flexible sigmoidoscopy)	Screening for primary prevention, initial cancer staging, strategies for surveillance post noninitial curative treatment
**Comparison; KQ 6**	Post-treatment surveillance strategies**:** variations in frequency and in modalities (e.g., CT, MRI, PET scans, biopsy, DRE, anoscopy, flexible sigmoidoscopy)	Screening for primary prevention, initial cancer staging, strategies for surveillance post noninitial curative treatment
**Outcomes; All KQs**	**Oncologic**: Overall survival, disease-specific survival, disease-free survival (including persistence, recurrence, or relapse), colostomy-free survival, local control, complete clinical response, salvage rate, sphincter preservation, health-related quality of life, treatment breaks (frequency or duration), treatment discontinuation, interruptions, or median treatment days, **Functional:** fecal or urinary incontinence, erectile dysfunction, sexual dysfunction, dyspareunia, use of vaginal dilators **Harms:** acute and late toxicity (hematologic, gastrointestinal, secondary malignancy, radiation dermatitis, radiation proctitis, radiation cystitis, pelvic insufficiency fractures, anal stenosis, vaginal stenosis)	
**Timing; All KQs**	No restrictions on duration of treatments or follow up.	
**Setting; All KQs**	Cancer care settings	
**Study Design; All KQs**	Randomized controlled trials, nonrandomized controlled trials, observational cohort with concurrent comparator, interrupted time-series, and other quasi experimental designs using appropriate analytic techniques.	Case reports, case series, commentaries, cross-sectional studies, reviews, qualitative studies, studies with sample size less than 30 patients (or less than 15 per treatment group/arm), nonrandomized studies with unspecified or poorly defined intervention/ treatment protocol (e.g., lack of names of chemotherapy agents used), nonrandomized studies with analytic techniques that don't allow drawing causal inferences.

Abbreviations: 3D CRT= three-dimensional conformal radiation therapy; IMRT= intensity-modulated radiation therapy; KQ= key question; MRI= magnetic resonance imaging; PET= positron emission tomography; DRE= digital rectal exam.

Supplementary Table 3. Interpretations of overall rating in strength of evidence assessment.

SOE Rating	Interpretation
**High**	We are very confident that the estimate of effect lies close to the true effect for this outcome. The body of evidence has few or no deficiencies. We believe that the findings are stable (i.e., another study would not change the conclusions).
**Moderate**	We are moderately confident that the estimate of effect lies close to the true effect for this outcome. The body of evidence has some deficiencies. We believe that the findings are likely to be stable, but some doubt remains.
**Low**	We have limited confidence that the estimate of effect lies close to the true effect for this outcome. The body of evidence has major or numerous deficiencies (or both). We believe that additional evidence is needed before concluding either that the findings are stable or that the estimate of effect is close to the true effect.
**Insufficient**	We have no evidence, we are unable to estimate an effect, or we have no confidence in the estimate of the effect for this outcome. No evidence is available, or the body of evidence has unacceptable deficiencies, precluding reaching a conclusion.

Supplementary Table 4. Study Characteristics

Study, Year	Study Location	Evaluable Patients, n	Intervention	Age, *y*	Sex (male), %	Race, %	HIV +, %	Tumor stage, %	Duration of follow-up	Risk of bias
LE vs. CRT										
Chai et al., 2018	United States	2,243	LE (n=503) vs. CRT (n=1,740)	†LE 54.5 vs. CRT 57.0	LE: 47.7<br>CRT: 32.3	LE:<br>White 83.3<br>Black 13.3<br>Other 2.0<br>CRT:<br>White 88.9<br>Black 8.4<br>Other 1.8	NR	LE<br>≤1cm: 41.6<br>>1 to ≤2cm; 36.6<br>Missing: 21.8<br>CRT<br>≤1cm: 21.5<br>>1 to ≤2cm: 53.5<br>Missing: 25.0	NR	Serious
Deshmukh et al., 2018	United States	190	LE (n=44) vs. RT alone (n=50) vs. CRT (n=96)	†LE 76 vs. RT alone 77 vs. CRT 74	NR	NR	Excluded	All were T1N0	NR	Critical
Gao et al., 2020	United States	883	LE (n=200) vs. CRT (n=683)	†Overall 60	Overall 35	Overall:<br>White 90<br>Black 8<br>Other 2	NR	Overall<br>≤1cm: 35<br>>1 to ≤2cm: 56<br>unknown 9	NR	Serious
RT alone vs. CRT with 5FU + MMC										
Bartelink et al., 1997	Netherlands	103	RT alone (n=52) vs. CRT (n=51)	RT alone: <60yrs (n=28)<br>CRT: <60yrs (n=28)	RT alone: 32.7<br>CRT: 25.5	NR	NR	T1-2N1-3: 15.5<br>T3-4N0: 46.6<br>T3-4N1-3: 35.9<br>TxNx: 1.9	Evaluated at 5 years	Moderate
ACT 1, 1996	United Kingdom	577	RT alone (n=285) vs. CRT (n=292)	†RT alone 65 vs. CRT 63	RT alone 47 vs. CRT 43	NR	NR	T1: 12.3<br>T2: 33.1<br>T3: 38.1<br>T4: 12.7<br>N+: 20.0<br>M1: 2.6	Evaluated at 3 years	Low
Northover et al., 2010	United Kingdom	577	RT alone (n=285) vs. CRT (n=292)	Reported in ACT1	Reported in ACT1	NR	NR	Reported in ACT1	Median 13.1 years	High
CRT with 5FU vs. CRT with 5FU + MMC										
Flam et al., 1996	United States	291	5FU (n=145) vs. 5FU+MMC (n=146)	‡60	35	NR	NR	T1: 15.1<br>T2: 38.1<br>T3: 37.5<br>T4: 9.2<br>N0: 82.5<br>N+: 17.5	Median 3.01 years	Moderate

CRT with 5FU + MMC vs. CRT with 5FU + cisplatin										
Ajani et al., 2008	United States	644	5FU+MMC (n=324) vs. 5FU+cisplatin (n=320)	‡55	30.7	NR	NR	T2: 64.6<br>T3: 26.4<br>T4: 9.0<br>N0: 69.6<br>N+: 25.9<br>Nx: 4.5	Median 2.51 years	Low
James et al., 2013	United Kingdom	940	5FU+MMC (n=472) vs. 5FU+cisplatin (n=468)	‡58	37.6	NR	Excluded	T1: 9.7<br>T2: 42.0<br>T3: 31.4<br>T4: 14.4<br>N0: 62.4<br>N+: 32.4<br>Nx: 4.7	Median 5.1 years	Low
Glynne-Jones et al., 2014	United Kingdom	884	5FU+MMC (n=472) vs. 5FU+cisplatin (n=468)	‡57	34.4	NR	Excluded	Reported in James et al.	Median 5.1 years	High
Gunderson et al., 2012	United States	649	5FU+MMC (n=325) vs. 5FU+cisplatin (n=324)	Reported in Ajani et al.	Reported in Ajani et al.	NR	NR	Reported in Ajani et al.	Up to 8 years	High
CRT with MMC + capecitabine + paclitaxel vs. CRT with MMC + capecitabine										
Gordeyev et al., 2022	Russia	144	MMC + capecitabine + paclitaxel (n=72) vs. MMC + capecitabine (n=72)	‡56.5	13.2	NR	NR	T1-2: 48.6<br>T3-4: 51.4<br>N0: 27.8<br>N+: 72.2	Median 39.5 months	Low
CRT with MMC + 5FU vs. MMC + cisplatin										
Matzinger et al., 2009	European Union	76	MMC + 5FU (n=39) vs. MMC + cisplatin (n=37)	‡55	30	NR	NR	T1: 1.3<br>T2: 47.4<br>T3: 38.2<br>T4: 11.9<br>N0: 51.3<br>N+48.7	Median 2 years	High
CRT with MMC + 5FU vs. MMC + capecitabine										
Jones et al., 2018	United Kingdom	147	MMC+5FU (n=95) vs. MMC + capecitabine (n=52)	<65 years: 58.5	29.3	NR	3.4	T1: 10.2<br>T2: 43.5<br>T3: 25.2<br>T4: 19.7<br>N0: 48.3<br>N+: 51.7	Evaluated at 1 year	Serious

								M1: 2.7		
Goodman et al., 2017	United States	107	MMC+5FU (n=63) vs. MMC + capecitabine (n=44)	‡59	27	White: 80<br>Black: 6<br>Hispanic: 11<br>Asian/Indian: 3	7.0	T1: 18<br>T2: 42<br>T3: 22<br>T4: 18<br>N0/Nx: 41	Median 49 months in 5FU group. Median 22 months in capecitabine group.	Serious
Peixoto et al., 2016	Canada	300	MMC+5FU (n=194) vs. MMC + capecitabine (n=106)	‡58	35	NR	4.0	T1-2: 66<br>T3-4: 34<br>N0: 55<br>N+:45	Median 43.9 months	Serious
IMRT vs. non-IMRT										
Pollom et al., 2017	United States	1165	IMRT (n=468) vs. non-IMRT (n=707)	‡70	35.5	White: 89.0<br>Non-white: 11.0	8.2	T1: 16.5<br>T2: 34.4<br>T3: 14.2<br>T4: 6.1<br>Tx: 28.8<br>N0: 71.0<br>Nx: 9.9	Median 47.4 months	Serious
Bryant et al., 2018	United States	779	IMRT (n=376) vs. non-IMRT (n=403)	†61	92.0	White: 83.2<br>Black: 13.9<br>Other: 2.9	20.4	T1: 17.5<br>T2: 49.0<br>T3: 27.9<br>T4: 5.6<br>N0: 68.8	Median 5.9 years	Serious
IMRT vs. 3D-CRT										
Dasgupta et al., 2013	United States	223	IMRT (n=45) vs. 3D-CRT (n=178)	‡59	34	White: 83<br>Other: 17	10	T1: 18.8<br>T2: 48<br>T3: 25.6<br>T4: 6.4<br>Tx: 1.4<br>N0: 61	Median 59.6 months	Critical
Elson et al., 2018	United States	6814	IMRT (n=2902) vs. 3D-CRT (n=3912)	‡59	30	White: 88<br>Black: 9.6	NR	T1: 10.7<br>T2: 50<br>T3: 38.5<br>T4: 0.8	Evaluated at 3, 5, and 10 years	Critical
IMRT with protons (IMPT) vs. IMRT with photons										
Mohiuddin et al., 2021	United States	208	IMPT (n=58) vs. IMRT (n=150)	†62	27	NR	12	T1: 16<br>T2: 52<br>T3: 24<br>T4: 7<br>N0: 50<br>M1: 2	Median 30 months	Serious

RT boost with EBRT vs. BT										
Moureau-Zabotto et al., 2013	France	99	EBRT (n=49) vs. BT (n=50)	†63	20	NR	NR	T1: 4<br>T2: 16<br>T3: 49<br>T4: 16<br>Tx: 14<br>N+: 100	Median 71.5 months	Serious
Hannoun-Levi et al., 2011	France	162	EBRT (n=76) vs. BT (n=86)	†65	29	NR	6	T1: 19<br>T2: 48<br>T3: 26<br>T4: 7<br>N0-1: 86<br>N2-3: 14	Median 62 months	Serious
Glynne-Jones et al., 2011	United Kingdom	424	EBRT (n=289) vs. BT (n=135)	NR (subset of ACT 1)	NR	NR	NR	NR	Median 13.1 years	Serious
Different doses of RT										
Peiffert et al., 2012	France	307	Standard; 60Gy (n=157) vs. High-dose; 65-70Gy (n=150)	†58.8	19	NR	NR	T1-2/N0-1: 37<br>N0-1: 76	Median 50 months	High
Tournier-Rangeard et al., 2008	France	119	All patients who filled out 2 questionnaires	<60 years: 55.5	21	NR	NR	T1-2: 43.5<br>T3-4: 56.5<br>N0: 60.9<br>N+: 31.9	Two months after treatment completion	High
Wegner et al., 2019	United States	7792	≤54Gy (n=4629) vs >54Gy (n=3163)	≤58 years old: 52	30	White: 89<br>Black: 9<br>Other: 2	NR	Stage I: 12<br>II: 45<br>III: 43	Median 42 months	Critical
Glynne-Jones et al., 2020	United Kingdom	931	5FU+MMC (n=472) vs. 5FU+cisplatin (n=468)	‡58	38	NR	NR	T1: 9.7<br>T2: 42.0<br>T3: 31.4<br>T4: 14.4<br>N0: 62.4<br>N+: 32.4<br>Nx: 4.7	Median 5.1 years	Critical
Dose-volume predictors of toxicity										
Lukovic et al., 2023	Canada	101	Subgroups had acute (n=87) and late (n=79) toxicity data	‡57	50.5	NR	25	T1: 11<br>T2: 55<br>T3: 28<br>T4: 7<br>N0: 64	Median 3.4 years	Critical
Nilsson et al., 2022	Sweden	114	Determination of dose-volume predictors of	‡63.7	21.9	NR	1	T1: 2.6<br>T2: 43<br>T3: 26.3	Median 40 months	Critical

			toxicity from the entire cohort					T4: 28.1<br>N+: 64.9		
Fractionation schema for RT										
Mehta et al., 2020	United States	6429	Determination of fractionation schema from the entire cohort	<60 years: 59.8	30	White: 80.6<br>Black: 8.4<br>Hispanic: 4.6<br>Other: 6.5	NR	T1: 14.9<br>T2: 51.8<br>T3: 24<br>T4: 8.7<br>Tx: 1.4<br>N0 62.6<br>Nx: 1.5	NR	Critical
One vs. Two cycles of MMC										
White et al., 2015	United States	217	One cycle (n=154) vs. two cycles (n=63)	‡60	30	NR	10.6	T1: 7.8<br>T2: 40.6<br>T3: 41.5<br>T4: 10.1<br>N0: 60.4<br>N+ 39.6)	Median 26 months	Serious
Post-treatment surveillance strategies										
Frazer et al., 2020	United States	138	High-risk group (n=61) vs. Low-risk group (n=77)	‡58	29	NR	14	T1: 19<br>T2: 39<br>T3: 29<br>T4: 11<br>N0: 59<br>N+: 41	Median 27 months	Critical

LE = local excision; RT = radiation therapy; CRT = chemoradiotherapy; NR = not reported; 5FU = 5-fluorouracil; MMC = mitomycin C; T = tumor; N = nodal; IMRT = intensity modulated radiation therapy; 3D-CRT = three-dimensional conformal radiation therapy; EBRT = external beam radiation therapy; BT = brachytherapy; Gy = gray

†Mean

‡Median

Supplementary Table 5. Findings for Comparisons with Insufficient Strength of Evidence

Intervention vs. Comparison	Outcome	Number of Studies and Design; Participants (n)	Findings
**LE vs CRT, early stage**	Overall survival	2 NRSIs; n=2433	No significant difference.<br>5-year, HR 1.06 (0.78-1.44, ref- CRT, n=2,243)<br>HR for CRT 1.74 (0.79-3.83; ref- LE; follow up time NR) HR for RT 2.17 (0.92-5.10; ref- LE; follow up time NR)
	Cause-specific survival	1 NRSI; n=883	No significant difference.<br>5-year, HR 0.48 (0.1-2.3)
**CRT with 5FU and MMC vs. 5FU alone**	Overall acute harms	1 RCTs; n=310	Favors 5FU.<br>Event rate: 5FU+MMC 14% (20/146) vs. 5FU 5% (7/144) (p<0.001)
	Acute hematologic toxicity	1 RCTs; n=310	Favors 5FU.<br>Event rate: 5FU+MMC 12% (18/146) vs. 5FU 2% (3/144) (p<0.001)
	Overall late harms	1 RCTs; n=310	No significant difference.<br>Event rate: 5FU+MCC 3% (5/146) vs. 5FU <1% (1/144) (p=0.26)
**CRT with 5FU + MMC vs. 5FU + cisplatin**	Colostomy-free survival	2 RCTs; n=1622	Conflicting evidence.<br>5-year cumulative colostomy rate: MMC 10% vs. cisplatin 19% (HR 1.68; 1.07-2.65; P=0.02).<br>HR 1.04 for cisplatin (0.82-1.31; ref- MMC).<br>Median follow up of 5.1 years.
**CRT with MMC + 5FU and MMC + cisplatin**	Progression-free and event-free survival, complete response, acute hematologic and gastrointestinal toxicity	1 RCTs; n=88	No significant differences. Comparisons listed MMC+5FU vs MMC+cisplatin<br>1 yr PFS, 76.3% vs 94.2%<br>1 yr EFS, 74.4% vs 89.2%<br>8 week CR rate, 59% vs 73%<br>Hematologic toxicity, 0% vs 24%<br>GI toxicity, 26% vs 27%
**CRT with MMC and 5FU vs. MMC and capecitabine**	OS, colostomy creation rate, distant metastasis, and locoregional recurrence rate	1 NRSI; n=107	No difference at 2 years. Comparisons listed MMC+5FU vs MMC+capecitabine.<br>OS, 87% vs 98%<br>Colostomy creation rate, 5% vs 9%<br>Distant metastasis, 14.7% vs 7.6%<br>LR recurrent rate, 6.5% vs 8.2%
	Colostomy-free survival	1 NRSIs; n=147	No significant difference at 1 yr.<br>5FU 90.7% vs capecitabine 77.5%
	Disease-free survival	2 NRSIs; n=397	No difference up to 5 yrs.<br>1 yr DFS, 5FU 79.3% vs capecitabine 76.2%<br>5 yr DFS, HR 0.99 (0.57-1.74, ref-capecitabine)
	Complete response	1 NRSI; n=100	No difference at 6 months.<br>5FU 91.4% vs capecitabine 88.1%
	Overall acute harms	1 NRSI; n=118	No difference at 6 weeks post-treatment.<br>5FU 55% vs capecitabine 45%
	Treatment break due to toxicity	1 NRSI; n=147	Favors capecitabine.<br>5FU 41% vs capecitabine 14% (p=0.006)
	Acute hematologic toxicity	2 NRSIs; n=225	Favors capecitabine at 6 weeks post-treatment.<br>5FU 27% vs capecitabine 4% (p<0.001)
	Acute dermatologic and gastrointestinal toxicity	2 NRSIs; n=225	No difference at 6 weeks post-treatment.<br>Dermatologic: 5FU 28% vs capecitabine 26%<br>GI: 5FU 13% vs capecitabine 17%

Intervention vs. Comparison	Outcome	Number of Studies and Design; Participants (n)	Findings
**IMRT vs. non-IMRT**	Overall and disease-specific survival	2 NRSI; n=1944	No significant differences. Listed IMRT vs non-IMRT.<br>2yr OS, 79.9% vs 79.5%<br>2yr DSS, 89.5% vs 85.7%<br>5yr OS, 74.5% vs 54.7%<br>5yr DSS, 87.3% vs 78.6%
	Tumor related colostomy-creation rate	1 NRSI; n=779	Favors IMRT.<br>IMRT 7% vs non-IMRT 12% (p=0.04)
	Acute grade 3+ hematologic toxicity	1 NRSIs; n=312	No difference at 90 days post-treatment.<br>IMRT 47% vs non-IMRT 40% (p=0.79)
	Acute grade 3+ bone marrow suppression	1 NRSIs; n=1165	No difference at 90 days post-treatment.<br>IMRT 9.8% vs non-IMRT 8.5%
**IMRT vs. 3D CRT**	Overall survival	2 NRSI; n=7037	Conflicting evidence. Listed IMRT vs 3DCRT.<br>2yr OS, 93% vs 90% (p=0.91)<br>10yr OS, 80.8% vs 76.7% (p=0.02)
	Disease-free, distant metastasis-free, and colostomy-free survival	1 NRSI; n=223	No difference over 2 years.<br>DFS, 87% vs 82% (p=0.20)<br>DMFS, 86% vs 88% (p=0.62)<br>CFS, 97% vs 91% (p=0.10)
**IMRT with protons vs. photons**	LRR and progression-free survival, acute and late grade 3+ overall toxicity	1 NRSI; n=208	No differences.<br>2yr LRR, 91% vs 88% (p=0.49)<br>2yr PFS, HR 0.6 (0.4-1.1, ref-protons)<br>Overall acute harms at 2 weeks post-treatment OR 0.7 (0.3-1.5)<br>Overall late harms >90 days post-treatment OR 0.8 (0.2-3.4)
**RT boost with EBRT vs. BT**	Overall and colostomy-free survival and LRR rates	1 NRSI (2 publications); n=162	No difference at 5 yrs. Listed EBRT vs BT.<br>5yr OS, 80% vs 78%, 5yr CFS 56% vs 71% (p=0.04), LRR 33% vs 12% (p=0.002)<br>5yr OS, 73% vs 76%, 5yr CFS 69% vs 74%, LRR 32% vs 11% (p=0.02)
	Overall, disease-specific, and relapse-free survival	1 NRSI; n=424	No difference over 13 yrs. Listed EBRT vs BT.<br>OS, HR 1.14 (0.81-1.6, ref- EBRT)<br>DSS, HR 1.16 (0.70-1.93, ref- EBRT)<br>RFS, HR 1.26 (0.91-1.75, ref- EBRT)
	Late ulcers/ radionecrosis	1 NRSI; n=424	Favors EBRT.<br>EBRT 6% vs BT 14% (p=0.003)
**Standard boost (15 Gy; total dose, 60 Gy)<br>vs.<br>high-dose boost (20-25 Gy; total dose, 65-70 Gy)**	Overall, colostomy-free, and disease-free survival, locoregional control, Quality of Life Questionnaire-C30, Anal Sphincter-Conservative Treatment questionnaire, and overall acute and late grade 3+ toxicity.	1 RCT; n=307	No difference. Listed as standard-dose vs high-dose.<br>5yr OS, 71% vs 74% (p=0.43)<br>5yr CFS, 74% vs 78% (p=0.07)<br>5yr DFS, 67.5% vs 70.6% (p=0.37)<br>5yr local control, 78.2% vs 83.1% (p=0.28)<br>Both QOL questionnaires had no numerical values reported but "no comparable differences"

Intervention vs. Comparison	Outcome	Number of Studies and Design; Participants (n)	Findings
**Dose, 45-54Gy vs. >54Gy**	Overall survival (up to 5 years)	1 NRSI; n=7792	Favors 45-54 Gy.<br>HR 1.10 (1.01-1.20, ref- 45-54Gy)
**Radiation therapy regimens:<br>group 1: 50.40Gy, 38-42 days (reference group) vs.<br>group 2: ≤40Gy;<br>group 3: >40Gy to <48.60Gy;<br>group 4: 50.40Gy, <38 days;<br>group 5: 50.40Gy, >42 days;<br>group 6: >52.20Gy**	Overall and progression-free survival	1 NRSI; n=931	Only listing significant results for overall survival at 3 years between groups due to multitude of comparisons. All comparisons with significant OS also had significant PFS.<br>Group 1 vs Group 2: Favors group 1<br>HR 8.24 (3.35-20.27)<br>Group 1 vs Group 3: Favors group 1<br>HR 3.12 (1.73-5.63)<br>Group 1 vs Group 5: Favors group 1<br>HR 1.72 (1.17-2.54)
**Small bowel V35Gy**	Grade 2+ acute diarrhea	1 NRSI; n=101	Significant predictor (p=0.03)
**Bladder D0.5cc**	Grade 2+ acute genitourinary toxicity	1 NRSI; n=101	Significant predictor (p=0.02)
**Anterior skin V35Gy**	Grade 2+ inguino-genital skin toxicity	1 NRSI; n=101	Significant predictor (p=0.02)
**Posterior skin V15Gy**	Grade 2+ perianal skin toxicity	1 NRSI; n=101	Significant predictor (p<0.01)
**Lower pelvis bone V45Gy**	Grade 2+ anemia	1 NRSI; n=101	Significant predictor (p=0.04)
**Large bowel V20Gy**	Grade 2+ late gastrointestinal toxicity	1 RCT; n=114	Significant predictor (p=0.04)
**≤4.7 fractions/week vs. >4.7 fractions/week**	Overall survival	1 NRSI; n=6429	Favors >4.7 fractions/week.<br>5yr OS, HR 0.70 (0.63-0.79, ref- <4.7fractions/week)
**Induction vs. no induction**	Overall, disease-free, colostomy-free, and disease-specific survival, Quality of Life Questionnaire-C30, Anal Sphincter-Conservative Treatment questionnaire and overall acute and late grade 3+ toxicity.	1 RCT; n=307	No significant differences.<br>5yr OS, 74.5% vs 71.0%<br>5yr DFS, 71.5% vs 64.8%<br>5yr CFS, 76.5% vs 75.0%<br>5yr DSS, 83.0% vs 78.5%<br>Both QOL questionnaires had no numerical values reported but "no comparable differences"
	Acute grade 3+ hematologic toxicity	1 RCT; n=307	Favors no induction over induction within 8 weeks of completing the boost.<br>Induction 29% vs no induction 12% (no p-value reported)

Intervention vs. Comparison	Outcome	Number of Studies and Design; Participants (n)	Findings
**Maintenance chemotherapy vs. none**	Overall, progression-free, colostomy-free, and disease-specific survival	1 RCT; n=940	No significant differences at 3 years.<br>OS, HR 1.07 (0.81-1.41, ref- maintenance)<br>PFS, HR 0.95 (0.75-1.21, ref- maintenance)<br>CFS, HR 0.87 (0.68-1.10, ref- maintenance)<br>DSS, HR 1.11 (0.80-1.54, ref- maintenance)
**1 vs. 2 cycles of MMC**	Overall, progression-free, colostomy-free, and disease-specific survival, and overall acute and late grade 3+ toxicity.	1 NRSI; n=217	No significant differences at 2 years. Listed 1 vs 2 cycles.<br>OS, 84% vs 91%<br>PFS, 78% vs 85%<br>CFS, 87% vs 92%<br>DSS, 88% vs 94%<br>Acute (<6 months) harms, 42% vs 41%<br>Late (>6 months) harms, 32% vs 22%
**Boost vs. no boost**	Overall, disease-specific, and relapse-free survival, and locoregional control	1 NRSI; n=577	No difference at median of 13.1 years follow up.<br>OS, HR 0.74 (0.48-1.15, ref- boost)<br>DSS, HR 0.62 (0.35-1.12, ref- boost)<br>RFS, HR 0.80 (0.52-1.22, ref- boost)<br>LRR, HR 0.90 (0.48-1.68, ref- boost)
	Late anorectal ulceration/ radionecrosis	1 NRSI; n=577	Favors no boost over boost.<br>Boost 8% vs no boost 0% (p=0.03)
**Event frequency: within 1 yr vs. within 2 yrs vs. year 3 to 5**	Frequency of total events, late grade 3+ toxicity, local recurrence, and distant metastasis	1 NRSI; n=138	No difference between annual versus twice a year frequency of surveillance beyond 2 years after initial treatment.

Abbreviations: LE- local excision; CRT-chemoradiation; 5FU- 5 fluorouracil, MMC-mitomycin C; RT- radiation therapy; RCT- randomized controlled trial; NRSI- non-randomized study of interventions; RR- relative risk; HR- hazard ratio; OR- odds ratio; NR- not reported; SOE- strength of evidence; IMRT- intensity modulated radiation therapy; 3D CRT- 3D conformal radiation therapy; EBRT- external beam radiation therapy; BT- brachytherapy; Gy-gray; OS- overall survival; DFS- disease-free survival; DSS- disease-specific survival; DMFS-distant metastasis-free survival; CFS- colostomy-free survival; PFS- progression-free survival; RFS- relapse-free survival; EFS- event-free survival; CR- complete response; GI-gastrointestinal; LRR- locoregional recurrence; QOL- quality of life.
